# Sternoclavicular joint septic arthritis in a healthy adult: a rare diagnosis with frequent complications

**DOI:** 10.5194/jbji-6-389-2021

**Published:** 2021-11-02

**Authors:** Rui Barbeiro Gonçalves, André Grenho, Joana Correia, João Eurico Reis

**Affiliations:** 1 Orthopaedics and Trauma Department, Centro Hospitalar Universitário de Lisboa Central, Lisbon, Portugal; 2 Cardiothoracic Surgery Department, Centro Hospitalar Universitário de Lisboa Central, Lisbon, Portugal

## Abstract

We report a case of complicated sternoclavicular joint septic arthritis in a previously healthy adult with no risk factors. An 83-year-old female presented to the emergency with a 1-week history of right shoulder pain followed by fever and prostration in the last 48 h. Computed tomography (CT) scan findings were consistent with right sternoclavicular joint (SCJ) septic arthritis complicated by periarticular abscess. Emergent surgical debridement was performed by a surgical team composed of orthopaedic and thoracic surgeons, followed by 6 weeks of antibiotic treatment. This case highlights the diagnosis and surgical treatment of a rare septic arthritis location but with frequent complications as well as the importance of multidisciplinary collaboration.

## 1 Introduction

Sternoclavicular joint (SCJ) infection is an uncommon condition, accounting for only 0.5 %–1.0 % of all septic arthritis and less than 0.5 % of septic arthritis in healthy patients (Tanaka et al., 2016). Several risk factors have been associated with SCJ infection, including intravenous drug use, concomitant infection at a different location, diabetes mellitus, trauma, and central venous line-related infection. Only 23 % of patients have no identifiable risk factor (Tanaka et al., 2016).

SCJ septic arthritis may lead to serious complications such as osteomyelitis, chest wall abscess, mediastinitis or myositis (Tanaka et al., 2016; von Glinski et al., 2019). Early diagnosis and immediate treatment are essential to diminish complications and improve outcomes. The most common causative pathogen is *Staphylococcus aureus*. Other pathogens include *Pseudomonas* spp., *Escherichia coli* and *Streptococcus* spp. (Ross and Shamsuddin, 2004; Kachala et al., 2016). Management usually consists of surgical debridement or en bloc resection and intravenous antibiotics (Kachala et al., 2016).

We report a case of an SCJ bacterial infection in a healthy adult with no identifiable risk factors.

## 2 Case presentation

**Figure 1:**
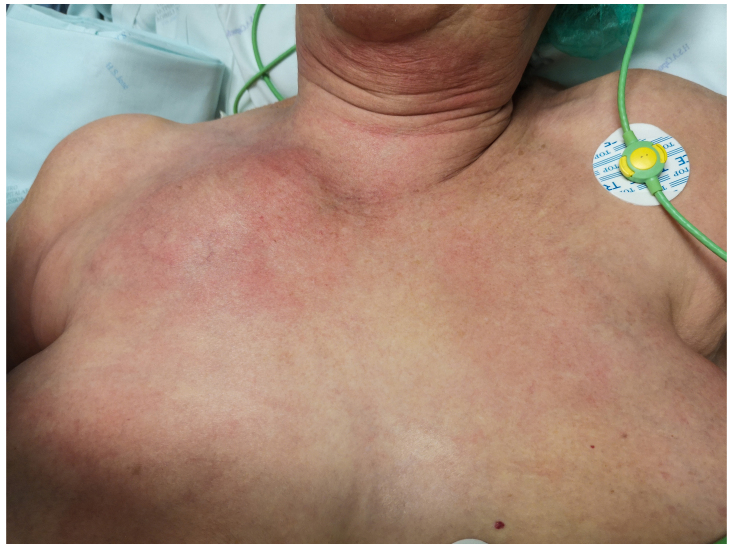
Clinical presentation. Swelling over the right SCJ with inflammatory signs.

**Figure 2:**
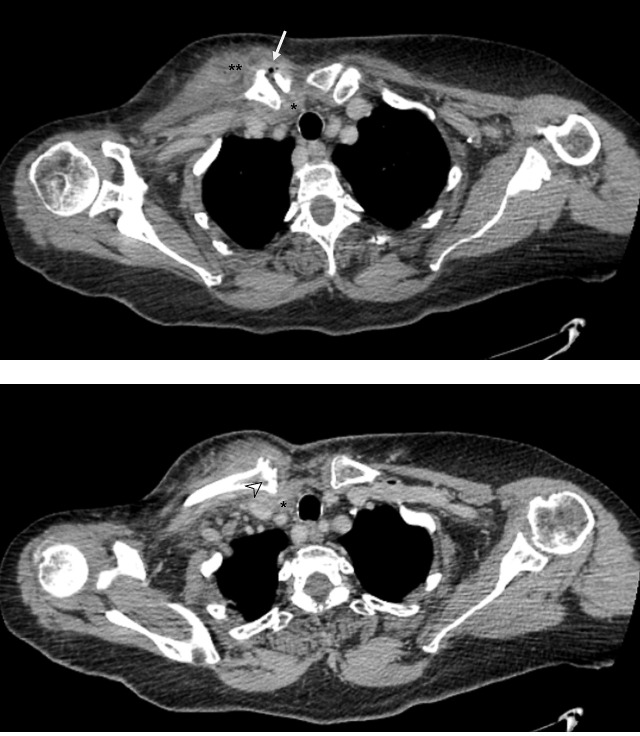
CT scan of the chest demonstrating signs of septic arthritis of the right SCJ, with articular space widening with gas formation, integrity and thickening of the SCJ capsule (arrow); medial clavicle bone erosion and cortical irregularity (arrowhead); periarticular abscess extending to the retrosternal space (*) and pectoral muscle (**).

An 83-year-old female presented to the emergency department with a 1-week history of right shoulder pain with radiation to the neck, followed by fever and malaise in the last 48 h. She had been previously diagnosed with shoulder tendonitis by her GP before installation of systemic symptoms. She was independent, active and otherwise healthy. She denied any history of trauma or other risk factors such as diabetes, drug abuse or recent infection. She was hemodynamically stable, and clinical examination revealed a warm painful swelling overlying the medial part of her right clavicle as well as erythema extending to the anterior chest wall (Fig. 1). Careful evaluation revealed no pain on isolated glenohumeral motion. White blood count was $16\times 10^{9}$/L (reference values (hereafter ref.) 4.5–$11\times 10^{9}$/L) with 80.6 % neutrophils (ref. 40 %–75 %), and C-reactive protein (CRP) was 381.3 mg/L (ref. <5.0 mg/L). Plain radiographs of the shoulder, chest and cervical spine revealed no significant findings. Computed tomography (CT) scan (Fig. 2) findings were consistent with right SCJ septic arthritis complicated by periarticular abscess with gas formation. Two blood cultures were taken, and the patient was admitted for surgical treatment.

**Figure 3:**
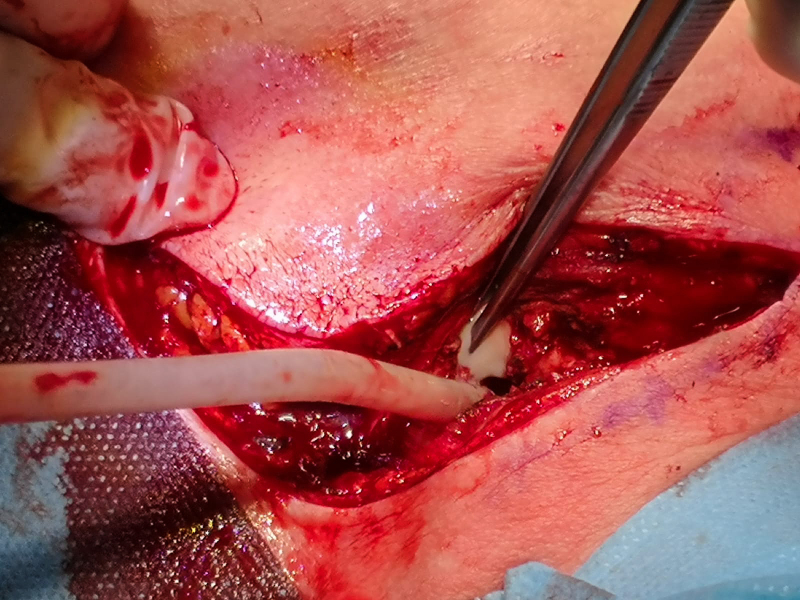
SCJ arthrotomy and evacuation of purulent material.

Emergent surgical debridement was performed by a surgical team composed of both orthopaedic and thoracic surgeons. The SCJ was approached through a J or “hockey-stick” incision over the medial clavicle and extended along the midline over the sternum. After arthrotomy, abundant purulent material (estimated 60 cc) was evacuated from the articular space, the pectoral fascia and the retrosternal space (Fig. 3). Pus and tissue samples were collected for cultures (aerobes, anaerobes and mycobacteria). Then, extensive irrigation (3 L of saline solution) and debridement of all necrotic tissue was completed and the SCJ capsule was not closed. Two closed-suction Blake^®^ drains were placed deep to the pectoralis and in the retrosternal space. The wound was primarily closed. Post-operative empirical antibiotic treatment was started with piperacillin/tazobactam, providing anaerobe coverage, as discussed with an infectious disease consultant. On day 4 post-op, *Streptococcus intermedius* was isolated in all six surgical samples and in one blood culture, and antibacterial therapy was tapered to Penicillin-G 24 M UI/d, which was continued for 6 weeks.

**Figure 4:**
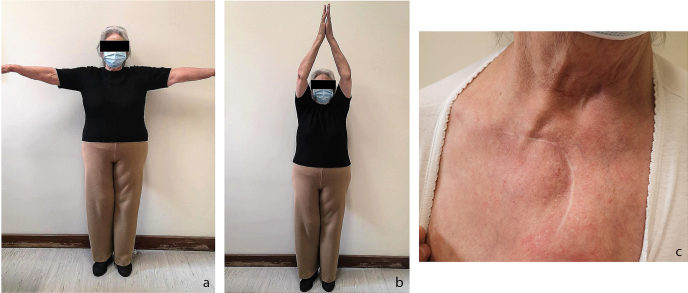
Clinical evaluation at 2 years' follow-up. Complete and symmetrical range of motion in abduction **(a, b)**. Cosmetic scar and mild reducible prominence of the medial clavicle **(c)**.

The patient remained afebrile in the post-operative period, with gradual improvement of the inflammatory signs and progressive normalization of laboratory findings. Immediate postoperative CT scan showed complete abscess removal and no recurrence was observed on repeated control CT at 2 weeks and 6 months after the surgical procedure. The patient was discharged from our outpatient clinic after a 2-year follow-up period. Clinical evaluation on the last visit revealed painless anterior reducible subluxation (positive medial piano-key sign) and a painless, symmetrical, full range of motion of the affected limb (Fig. 4), with a Constant score of 80.

## 3 Discussion

SCJ infection accounts for less than 1 % of all bone and joint infections (von Glinski et al., 2019; Bar-Natan et al., 2002) and is rare in the healthy population. In their literature review from 2002, Bar-Natan et al. found only 27 documented cases of SCJ infection in healthy individuals (Bar-Natan et al., 2002). Recently, in 2019 von Glinski et al. (2019) found that diabetes mellitus was the most common risk factor (61.5 %), followed by glucocorticoid medication (30.8 %) and intravenous drug abuse (two patients).

The lack of awareness and the relatively unspecific clinical findings can delay diagnosis and treatment initiation. In fact, SCJ septic arthritis often presents with insidious onset of vague, poorly localized symptoms and a lack of systemic complaints. The strong surrounding ligaments that reinforce the SCJ itself create a relatively non-distendable space. As a result, SCJ effusions progress slowly, with large effusions appearing late. The median duration of symptoms at presentation is approximately 14 d (Ross and Shamsuddin, 2004), contributing to the increased prevalence of complications.

As with our patient, referred pain (e.g., shoulder and/or neck pain) is a frequent presenting symptom and can be misleading. A sound knowledge of clinical anatomy and an adequate clinical examination is essential for localizing the problem and raising suspicion (Fry and Boyle, 2013).

Radiologic studies are usually taken as part of the diagnostic investigation. Plain X-rays are usually normal, but signs of SCJ pathology can be present, such as sclerosis of the medial end of the clavicle, subchondral bone erosion, soft tissue swelling or periosteal reaction. CT scan should be routinely performed in cases of suspected SCJ infection, both for diagnosis and identification of complications. In a review of 180 cases, the authors found that all the 95 scans performed demonstrated at least one abnormality, including osteomyelitis (69 %), chest wall abscess or phlegmon (57 %), joint space widening or fluid (25 %), mediastinitis (20 %) and extra-pleural abscess (1 %) (Ross and Shamsuddin, 2004). In their study, von Glinski et al. (2019) performed CT scans on all patients and found even higher prevalence of abscess formation (76.9 %).

Definitive diagnosis is established by microbiological studies provided by needle aspiration, blood cultures, and surgical specimens. Needle aspiration may be attempted when the diagnosis is in question, but the paucity of SC articular fluid and the presence of an intra-articular disk hinder the procedure and limit the success rate (50 % to 77 % of cases) (Ross and Shamsuddin, 2004; Womack, 2012). Tissue cultures of surgical specimens are the most reliable source of agent isolation (96 % of cases) (Kachala et al., 2016). In similarity to septic arthritis in other joints, *Staphylococcus aureus* is the most frequent agent in the vast majority of published cases. In a retrospective single-institution study from 2016, Kachala et al. (2016) obtained *Staphylococcus aureus* isolation in 58 % of patients, methicillin-resistant *S. aureus* (MRSA) in 5 %, *Streptococus* spp. in 10 %, Gram-negative species in 8 %, and anaerobes in 8 % (Kachala et al., 2016). The *Streptococcus intermedius* isolated in our patient is a β-hemolytic Gram-positive member of the *Streptococcus milleri* group. It is commonly associated with abscess formation, as observed in our patient. We admitted haematogenous aetiology for this infection, although the source has not been identified.

Management of SCJ septic arthritis consists of surgical debridement and intravenous antibiotics. Conservative measures are frequently ineffective since extra-articular complications are usually present at the time of diagnosis. In their review, Ross and Shamsuddin (2004) reported medical management in 42 % of cases, with a failure rate of 15 %. Because of a high recurrence rate and persistent latent infection reported in some studies, some authors have been proposing a more aggressive approach, with formal joint resection, including medial clavicle, lateral sternal manubrium and the first and second ribs (Kachala et al., 2016). This approach is supported by good functional outcomes, as the resulting joint instability is generally well tolerated (von Glinski et al., 2019). The limited resection in our patient has, however, been successful in eradicating infection, as evidenced by the clinical results and the 2-year control CT scan. It is, in our opinion, a less aggressive approach, possibly providing a faster recovery and a better functional outcome. Other reports in the literature have observed good results with this approach (Tanaka et al., 2016; Thompson and Barlotta, 2018; Grunspun et al., 2008).

Evidence on which to base empiric antibiotic treatment or its duration is limited. It usually ranges from 4 weeks in uncomplicated SCJ arthritis up to 6 weeks in cases where complications are observed (Ross and Shamsuddin, 2004). *Staphylococci* coverage is essential. The high prevalence of MRSA in some populations must be considered, and some specific risk factors like intravenous drug abuse and dialysis justify the use of vancomycin (von Glinski et al., 2019). One recent randomized controlled trial has shown that oral antibiotic therapy was non-inferior to intravenous antibiotic therapy in the treatment of orthopaedic infection (Li et al., 2019). However, in complicated SCJ infection, the risk of systemic spreading and the proximity to the adjacent mediastinal structures warrant the use of an intravenous scheme and longer duration. We believe that antibiotic selection and duration should always be discussed with an infectious disease consultant and based on local protocols and resistance patterns.

## 4 Conclusion

SCJ septic arthritis is a rare infection, especially in healthy adults with no risk factors. Diagnosis is often delayed, leading to frequent extra-articular complications. We emphasize the need for an appropriate history and precise clinical examination to localize symptoms and raise suspicion of SCJ arthritis. CT scan is essential for diagnosis, identifying complications and treatment planning. Surgical drainage and aggressive debridement is the mainstay of treatment, followed by intravenous antibiotic therapy. Multidisciplinary collaboration (orthopaedics, thoracic surgery and infectious disease) is recommended for better outcomes.

## Data Availability

The data that support the findings of this study are available from the corresponding author, Rui Barbeiro Gonçalves, upon reasonable request.
